# Indicators Related to Marital Dissatisfaction

**DOI:** 10.3390/healthcare11131959

**Published:** 2023-07-07

**Authors:** Claudia Sánchez, Cecilia Mota

**Affiliations:** 1 Coordination of Psychology Research, National Institute of Perinatology (INPer), Mexico City 11000, Mexico; clausanbra@yahoo.com; 2 National Institute of Perinatology, Research Tower, 1st Floor, Lomas Virreyes, Mexico City 11000, Mexico

**Keywords:** marital dissatisfaction, intimate partner violence, self-esteem, coping, gender roles

## Abstract

This is a study on indicators related to marital dissatisfaction. The research was conducted by the psychology department of a reproductive health institution in Mexico City. The objective was to know the relation between marital satisfaction/dissatisfaction and gender roles, self-esteem, the types of coping strategies and the types of violence perceived from the partner. It was a nonexperimental, retrospective, cross-sectional study of two samples—one of women and one of men—classified by marital satisfaction or dissatisfaction. The nonprobability quota sampling included 208 participants: 104 women and 104 men. Comparisons, correlations and a discriminant analysis were made to identify the most significant variables. Women with marital dissatisfaction perceived blackmail, psychological violence and humiliation/devaluation from their partner; they preferably adopt a submissive gender role and use escape/avoidance as a coping strategy, and so do the men with marital dissatisfaction, who also perceived blackmail, control and psychological violence from their partner; they have low self-esteem, and they preferably adopt a submissive gender role. Isolating factors will allow for more specificity in terms of psychological care at health institutions as well as avoiding gender biases and preventing an increase of violence in couples.

## 1. Introduction

Marital satisfaction is related to behaviours that provide well-being and produce the ability to make agreements and solve problems in couple interactions [1,2,3]. In contrast, marital dissatisfaction has a negative impact on the quality of life, health and job satisfaction of people who live with it [4,5]; furthermore, it is a risk factor of domestic violence [6], which, to a greater extent, affects people who live in a couple relationship, their family and their surroundings [7,8,9,10]. 

Studies carried out in the Mexican population have indicated that both men and women believe couple relationships should be equitable for them to be satisfactory, and that they must communicate and solve their problems for the relationship to improve [11,12]. Other research on the topic has found several factors that are related to marital dissatisfaction; amidst the most noted ones are domestic violence, gender roles, low self-esteem and the types of coping.

The presence of domestic violence, defined as “an act or omission whose purpose is to hurt or wound another person, violating their rights” [13], p. 29, has been linked to a higher incidence of marital dissatisfaction in both women and men. Studies on heterosexual women show a high rate of psychological violence exerted by their partners, this being more frequent than physical violence [14], affecting their mental health and causing marital dissatisfaction [15]. Furthermore, it has been mentioned that domestic violence experienced mainly by women and girls has gone from being a hidden and tolerated event to a public health problem of a legal nature [13]; nevertheless, there are some studies that indicate that heterosexual men are also victims of violence by their partners, but it is less reported and has been made invisible due to cultural matters [16].

Gracia [17] noted that the reports of domestic violence show only a small percentage of the seriousness of this issue, with the added difficulty of it possibly turning into an actual lifestyle rather than being an isolated event. An example of this is shown in research on men who were victims of violence, where it was found that, due to the education they received and their social constructs, they did not have the ability to set boundaries, thus normalising the abuse exerted by their partner and creating marital dissatisfaction [18]. In this way, the presence of violence exerted by the woman towards her partner questions the notion that the woman is always the victim and that the man is always the abuser [19]. Likewise, a relation between other forms of violence—such as psychological violence—and marital dissatisfaction has been found [20,21,22,23]. In this regard, it is worth noting that psychological violence implies neglect, abandonment, infidelities, threats, insults, humiliations and the restriction of self-determination and decision-making power, all of which have an impact on self-esteem and produce feelings of deprecation and death wishes [24].

On the other hand, physical violence implies wounding the other person’s body by means of physical strength with an object or weapon [8,24,25,26,27], which can last for years [28]. A study on Nigerian women experiencing marital dissatisfaction showed that physical and sexual violence exerted by their partners increased in those who had paid employment [29,30], whereas a similar study in the United States found that a decrease in pay gap reduces marital dissatisfaction and intimate partner violence [31].

Gender roles play an important part in couple relationships. Even when they are the product of socially established stereotypes for each gender [32], they may coexist in every person regardless of them being a man or a woman [33]. Furthermore, the distribution of both traditional and modern gender roles in a couple is influenced by their sociocultural context.

In contrast, the rigidity of gender roles in couple interaction increases marital dissatisfaction [11,34,35]. Shechory et al. [36] found that submissive women consider their marital life unequal, and they manifest poor sexual and marital satisfaction [37,38]. Likewise, Cazes [39] found that a patriarchal relationship still prevails in many couples in Mexico, where the woman must fulfil the traditional role assigned by society (maternity, house chores, etc.) even though the roles performed by both men and women have now evolved.

When diminished, self-esteem may also affect couple dynamics, producing marital dissatisfaction, for both women and men usually have self-deprecating responses [40]. Studies conducted in different populations have found relations between low self-esteem and marital dissatisfaction; for example, in violent Mexican indigenous women, a relationship has been found between low self-esteem and marital dissatisfaction [41]. Murray et al. [42] showed some differences in the perception of marital satisfaction between people with low self-esteem and people with high self-esteem. The former perceive that the issues in their relationship indicate a lack of affection towards them, causing them to respond with disdain and distance, thus generating dissatisfaction; on the other hand, the people with high self-esteem are less sensitive to problems and reaffirm their relationship by feeling satisfied. Another study where romantic relationships were analysed found a positive correlation between high self-esteem, happiness and couple satisfaction [43]. Aguilar et al. [44] compared the self-esteem of 48 abused women with 48 non-abused women and found that abused women show lower self-esteem than non-abused women, and that their relationships suffer from emotional abuse, impotence and hopelessness, all of which produce marital dissatisfaction.

Another factor related to marital dissatisfaction is the types of coping, which is a moderator between stressful events and the regulator of the emotional response to a problem [45,46,47,48]. It has been found that chronic stress appears when there is a lack of balance between the demands of the surroundings and the means to face them [49,50]. A study conducted in women with marital dissatisfaction and domestic violence revealed that the women with better coping strategies managed to better face this problem when compared to those who had less adaptive strategies [51]. Additionally, Puente-Martínez [52] compared the strategies of emotional regulation that were used by 200 women with marital dissatisfaction who survived intimate partner violence; the results indicated that they were passive at the beginning and used more active strategies later, which in turn helped them to end the abuse and dissatisfaction in their relationships.

Studying the factors that intervene in marital dissatisfaction and being able to isolate the variables that contribute to a better understanding of this interaction allows for the creation of more specific and efficient psychological intervention strategies.

Hence, the objective of this work was to study the relation between marital satisfaction/dissatisfaction, the type of violence perceived from a partner, gender roles, self-esteem and the types of coping used by a sample of Mexican women and men who visited a reproductive health institution to obtain indicators for a more specific psychological intervention.

## 2. Materials and Methods

A retrospective cross-sectional study with a multivariate, correlative, comparative design was conducted with two independent samples (one of women and one of men), each of them stratified according to the score they obtained on the scale for marital satisfaction/dissatisfaction, which was the classifying variable. It must be noted that the sample of men was selected by making sure there was an equivalence with the sample of women in terms of the control variables so both samples could have similar characteristics given that the subjects were not couples.

### 2.1. Participants

With an intentional nonprobability quota sampling, the samples were recorded during one year as stipulated in the project. The sample was composed of 208 participants, 104 women and 104 men, who entered the National Institute of Perinatology (Instituto Nacional de Perinatología, INPer) for medical care. The samples were recorded and analysed independently and not as couples. The inclusion criteria were: men and women of legal age, with minimum primary schooling, a one-year minimum relationship and no prior diagnosis of mental retardation or psychotic disorders. The controlled sociodemographic factors were: age, marital status, schooling (measured in years), occupation and the motive for visiting the INPer, which in the case of the women could be either obstetrical (pregnancy control) or gynaecological (any reproductive problem). As for the men, they did not have a medical diagnosis because they were only keeping a relative company who did have a medical appointment.

### 2.2. Procedure

The participants who met the inclusion criteria were given an identification sheet, and the application of the instruments was carried out in a single session before receiving any type of medical or psychological care. As part of their comprehensive treatment, psychological care was offered by the psychology department.

### 2.3. Ethical Aspects

The project was approved by the institutional research and ethics committees, with the following registration number: 212250-3110-10810-02-16. The participants signed the informed consent form, where it was specified that their data are anonymous and confidential.

### 2.4. Classification Variables

Sex and marital satisfaction or marital dissatisfaction.

### 2.5. Intervening Variables

Intimate partner violence, gender roles, self-esteem and types of coping.

### 2.6. Instruments

It must be noted that the psychometric indexes were taken from the original instruments that were validated for the Mexican population because, given the size of the sample, validations could not be conducted for the population of the study.

#### 2.6.1. Multifaceted Inventory of Marital Satisfaction

It evaluates aspects of the couple’s marital life with 85 Likert statements, validated for the Mexican population, with a Cronbach’s alpha of 0.97 for internal consistency; the results were classified according to the scores obtained either above or below the cutoff point (188) [53].

#### 2.6.2. Scale of Violence

It measures eight types of violence perceived in couples: physical, economic, intimidation, psychological, control, humiliation/deprecation, blackmail and sexual; it consists of 39 Likert test items validated for the Mexican population; the Cronbach’s alpha for reliability was 0.97 [54].

#### 2.6.3. Masculinity-Femininity Inventory (IMAFE)

It is a Likert scale that measures gender roles and is made of 15 test items per dimension (femininity, masculinity, machismo and submission). It is based on the most representative aspects of the gender roles and stereotypes found in Mexican culture. It was validated for the Mexican population, and the obtained Cronbach’s alpha for reliability was 0.92 [55].

#### 2.6.4. Coopersmith Self-Esteem Inventory (CSEI)

Validated for the Mexican population with a Cronbach’s alpha of 0.81, it yielded two intervals: low level (less than 17) and normal level (18 to 23 points); it is made of 25 test items [56].

#### 2.6.5. Coping Scale

It is made of 67 Likert test items and measures eight types of coping: Confrontational: direct actions to alter the situation; Distancing: efforts to remove oneself from the situation; Self-control: efforts to control feelings and actions; Social support: seeking support; Responsibility: acceptance of responsibility; Escape/avoidance: avoiding the problematic situation; Problem solving: efforts to change the situation with a reflective approach; Positive re-evaluation: creating a positive meaning based on personal resources. It was validated for the Mexican population with a Cronbach’s alpha of 0.85. The highest score will be the ranking to be assigned [47,50,57].

### 2.7. Description of Samples

The final sample was made of four groups: women with and with no marital satisfaction (group 1 and group 2), and men with and with no marital satisfaction (group 3 and group 4). Measures of central tendency and dispersion were applied for the description of the controlled sociodemographic factors, and for the classification of marital satisfaction or dissatisfaction, x2 and Student’s *t*-test were applied. For the analysis of the variables, Student’s *t*-test and Pearson’s product-moment correlation test were applied. A discriminant analysis of the significant variables was performed to find the linear combination of the most significant variables to differentiate the groups. The analysis was performed with SPSS-22 software. The Student’s *t*-test and the Pearson product-moment correlation test were applied to analyse the variables. A discriminant analysis of the significant variables was carried out to find the linear combination of the most significant variables to differentiate the groups. The analysis was conducted with the software SPSS-22.

### 2.8. Controlled Sociodemographic Factors

The characteristics of the samples, where some of the participants were a couple, were captured and worked on independently; however, this contributed to the similarity of the samples, which are shown in Table 1.

Regarding occupation, women were distributed as follows: 77.9% (81) were housewives, 8.7% (9) were employees, 7.7% (8) were underemployed (informal jobs) and 5.8% (6) were professionals (they practise a specialised academic profession). In men, the distribution was as follows: 58.7% (61) were employees, 28.8% (30) were underemployed and 12.5% (13) were professionals.

## 3. Results

Regarding the classification of marital satisfaction and marital dissatisfaction in the group of 104 women, 43 (41.3%) reported being satisfied in their relationship (group 1), and 61 (58.7%) expressed being unsatisfied (group 2). In the group of men, 58 (55.8%) reported being satisfied (group 3), whereas 46 (44.2%) said they were not (group 4) (see Table 2).

Regarding the results through the *t*-test between women and men with marital satisfaction, women presented significantly lower scores than men (see Table 3).

In the results of the study variables classified by gender and by marital satisfaction or dissatisfaction, the following results were obtained.

In women, statistically significant differences were found between those satisfied (group 1) and those unsatisfied (group 2) in terms of gender dimensions: femininity, masculinity and submission. Femininity and masculinity turned out to be related to marital satisfaction, whereas submission was related to dissatisfaction. Regarding self-esteem, statistically significant differences were also observed between groups 1 and 2, with higher scores found in group 1.

As for the types of coping, significant differences between group 1 and group 2 were only found in escape/avoidance, which was related to marital dissatisfaction. Problem solving and positive re-evaluation were related to marital satisfaction in spite of their marginal significance (see Table 4).

Furthermore, in terms of the types of violence, significant differences between group 1 and group 2 were found for all types of violence in relation to marital dissatisfaction with the exception of physical violence (see Table 5). It must be noted that the effect sizes in the significant variables went from low to medium.

In the sample of men, statistically significant differences were observed between those who were maritally satisfied (group 3) and those who were not (group 4) in terms of gender dimensions: femininity was related to satisfaction, and machismo and submission were related to dissatisfaction (see Table 6).

The high self-esteem scores were also related to satisfaction, and, regarding the types of coping, escape/avoidance turned out to be related to dissatisfaction, while problem-solving was related to marital satisfaction (see Table 6). As for the types of violence, statistically significant differences were also found between groups 3 and 4, with all the types of perceived violence being related to marital dissatisfaction (see Table 7). The effect size in men was medium.

Correlations were made in both women and men between the studied variables and marital satisfaction/dissatisfaction with the purpose of identifying the most significant variables (Table 8 and Table 9).

The discriminant analysis was performed with the significant variables in women and men. For the women, a function with 13 variables was obtained, explaining 100% of the differences and the variance between satisfied and unsatisfied women, an eigenvalue of 0.611, a Wilks’ lambda of 0.621, and a canonical correlation of 0.616 with a significance of *p* ≤ 0.001, which allowed the discrimination of the variables related to marital satisfaction or dissatisfaction. The standardised coefficients showed that violence is the variable that most contributes to marital dissatisfaction, particularly that of blackmail (0.639); it is followed by psychological violence, humiliation/devaluation, economic violence (controlling someone through money), intimidation, control and sexual violence; they preferably settle for a submissive gender role and use escape-avoidance as coping. By contrast, women with marital satisfaction showed an adequate level of self-esteem, a preference for feminine or masculine gender roles, and positive re-evaluation as their type of coping. The centroids showed –0.922 for satisfied women and 0.650 for unsatisfied women. Therefore, it can be concluded that women with and with no marital satisfaction have specific indicators related to this condition in 76.0% of the correctly classified cases; see Table 10.

For the men, the discriminant analysis was performed with the significant variables; a function with 13 variables was obtained, explaining 100% of the differences and the variance between satisfied and unsatisfied men, an eigenvalue of 0.808, a Wilks’ lambda of 0.553, and a canonical correlation of 0.669 with a significance of *p* ≤ *0*.001, which allowed the discrimination of the variables related to marital satisfaction or dissatisfaction. The standardised coefficients showed that men with marital dissatisfaction perceive control-type violence from their partner, which is the variable that most contributes to marital dissatisfaction with 0.728; it is followed by blackmail, psychological violence, economic violence, humiliation/devaluation, sexual violence, intimidation and physical violence; they preferably settle for a submissive gender role and use escape/avoidance as coping. By contrast, men with marital satisfaction showed an adequate level of self-esteem, a preference for feminine gender roles, and problem solving as their type of coping. The centroids showed -.793 for satisfied men, and 1.000 for unsatisfied men. Hence, it can be concluded that men with marital dissatisfaction have specific indicators related to this condition in 84.6% of the correctly classified cases (see Table 11).

## 4. Discussion

The objective of this paper was to study the relation between marital satisfaction/dissatisfaction, the type of perceived violence, gender roles, self-esteem and coping styles in a sample of Mexican women and men. One of the early findings was the differences between women and men in terms of the percentages for marital satisfaction/dissatisfaction; the percentage of unsatisfied women is 14.5% greater than that of unsatisfied men, which shows a disadvantage for women.

A second finding was the indicators associated to both marital satisfaction and dissatisfaction. Regarding those related to marital dissatisfaction, some similarities were found between both sexes.

The first indicator associated to marital dissatisfaction in both women and men was the perceived violence; the difference lies in the type of violence exerted on the other. In women, the most important factor was perceiving blackmail from their partner, followed by psychological abuse and humiliation/devaluation; in men, it was the perception of their partner exerting control over them, followed by blackmail, psychological and economic abuse. This coincides with Moral et al. [58], who stated that when conflicts are faced inadequately, these become chronic, leading to fights, distancing, indifference and, finally, to violence.

These results differ from what has been noted by other research carried out in Mexico where women are emphasized as the victims of marital violence [59,60]. Possibly, the difference between results is due to the way people tend to give socially desirable answers marked by gender prejudices in massive surveys. Nevertheless, couple violence has been studied in other Latin American countries where similar results to the ones obtained in this study have been found [61].

This would indicate that it cannot be stated that the man is the only one exerting violence in a couple, for many of these examples of violence are focused on the woman as the victim of the man [23,62,63]; therefore, it is important to do research with both sexes to widen the scope of the problem.

However, it must be noted that the studied population for this research comes from the general Mexican population because the INPer is not an institution specialised in women who were victims of violence with a prevalence of physical violence, where different factors could be found.

The second indicator related to marital dissatisfaction has to do with the gender roles established by couple dynamics; the results showed that submission prevailed as a characteristic in both maritally unsatisfied women and men. This coincides with a study on women and the relation between emotional dependency and intimate partner violence, where a high relation was found between the presence of both conditions in couples, resulting in attitudes of subordination and submission [64].

Low self-esteem was the third indicator found in women and men with marital dissatisfaction, which coincides with Echeburúa [65], who found that men with low self-esteem felt unsatisfied in their relationship and showed high levels of jealousy, possessiveness, irritability towards boundaries and poor impulse control. People with low self-esteem frequently struggle with self-confidence; when it comes to marriage, this insecurity leads them to behave in a way that fosters distancing, violence and dissatisfaction instead of contributing to a satisfactory couple dynamic.

The escape/avoidance type of coping was the fourth indicator related to marital dissatisfaction in both women and men; it is translated as avoiding conflict. Méndez and García [66] also found that this type of coping is a variable that predicts several types of violence that generate dissatisfaction in a relationship. This behaviour emphasizes marital dissatisfaction because it prevents both partners from facing conflicts and modifying some of their elements. Behaviours such as indifference, the silent treatment and not taking any actions to solve problems contribute to dissatisfaction, and they are risk factors that lead to violence [67].

In contrast, the first indicator related to marital satisfaction in both women and men was high levels of self-esteem. Our results showed that women and men who scored high levels of marital satisfaction also had high self-esteem.

A possible explanation for this is that people who trust their abilities and have a positive image of themselves are able to establish effective communication with their partner, express their needs and wishes in a clear way, and set healthy boundaries. They also tend to be less critical of themselves and their partner, which helps to avoid unnecessary conflicts [68].

In second place, we found that marital satisfaction is related to femininity and masculinity in women, for they involve demonstrations of affection and the care for others as well as self-affirmation. Likewise, in maritally satisfied men, femininity was the one prevailing characteristic.

The last indicator related to marital satisfaction in women was positive re-evaluation as a type of coping, which is a strategy centred on the control of emotion when facing a stressful situation, giving it a positive meaning that functions as an adaptive resource. In men, the prevailing type of coping was that of problem-solving, which consists of making an effort to change a stressful situation by means of reflection and assertive behaviour.

The found indicators allow us to better steer the psychological intervention as referenced by Santelices [69], who said that intervention models will help focus the factors related to couple conflicts to avoid damage that has an impact on the family at the expense of their psychological, physical and labour well-being.

As can be observed, some indicators were isolated in this study to provide guidelines for the psychological intervention in people with couple problems.

## 5. Limitations

One of the main limitations was the small size of the sample, hence the use of psychometric indexes and cutoff points from the original instruments that were validated for the Mexican population, which limits the generalisation of the results.

Another limitation was that it only measured perceived violence and not exerted violence; in a population that suffers from exerted violence, the profiles will probably differ. One further limitation is that it is a nonprobability sample, and no generalisations can be made, for the results only can show risk indicators for populations with similar characteristics.

It should be noted that another limitation is that it was not a couple study; these results are from women and men with and without marital satisfaction but who were worked with independently. For future research, it would be important to carry out a study of dependent samples where the wife/husband pairing is used.

## 6. Conclusions

In this study, marital dissatisfaction is 14.5% higher in women; however, generalisations cannot be made, since this study was carried out on a non-random sample in a population with particular characteristics.

The violence perceived from the partner is the same in both groups with marital dissatisfaction.

Marital dissatisfaction is related to submissive characteristics in both sexes.

A decrease in self-esteem is a factor related to couple conflicts.

The type of coping that most contributes to marital dissatisfaction in both sexes is escape/avoidance.

The generation of indicators in different populations by isolating factors that explain the complexity of couple conflicts with no gender biases will contribute to the creation of psychological intervention strategies with greater specificity to avoid the worsening of these conflicts that affect not only both members of the couple but also their surroundings. This work is an incursion in couples who have relationship problems. By isolating explanatory factors, other aspects must be explored in different populations to gain a better understanding of the complexity of couple dynamics.

## Figures and Tables

**Table 1:** Characteristics of the sociodemographic factors of the samples.

Variables	Women n = 104	Men n = 104
Age	32.3 ± 6.14 Range: 22 to 56 years old	35.1 ± 8.04 Range: 22 to 59 years old
Schooling	12.3 ± 3.23 years	13.0 ± 3.58 years
Married	64 (61.5%)	64 (61.5%)
Single	1 (1%)	6 (3.4%)
Civil union	39 (37.5%)	39 (37.5%)
Obstetrical	40 (38.5%)	40 (38.5%)
Gynaecological	64 (61.6%)	64 (61.6%)
Relationship/partner average	7.1 ± 5.2	7.1 ± 5.2

**Table 2:** Differences between marital satisfaction and dissatisfaction by gender.

	With Marital Satisfaction	With Marital Dissatisfaction	Total	x^2^	*p*
Women	43 (41.3%)	61 (58.7%)	104		
Men	58 (55.8%)	46 (44.2%)	104	4.33	0.05 *

* *p* ≤ 0.05.

**Table 3:** Comparison between marital satisfaction and dissatisfaction by gender.

	Marital Satisfaction n = 104 Mean DS	t value	Sig.
Women	3.87 ± 0.66	2.201	0.02 **
Men	4.06 ± 0.56		

** *p* ≤ 0.01.

**Table 4:** Differences and relation between women with and with no marital satisfaction and their gender role, self-esteem and type of coping.

	Group 1 with Marital Satisfaction n = 43 Mean SD	Group 2 with Marital Dissatisfaction n = 61 Mean SD	*t* Value	Sig.	ƞ
Femininity	5.48 ± 0.97	4.77 ± 0.99	3.63	0.000 ***	0.34
Masculinity	4.69 ± 0.88	4.23 ± 0.92	2.57	0.012 **	0.24
Machismo	2.90 ± 0.85	3.20 ± 0.93	−1.68	0.09	
Submission	2.37 ± 0.65	2.87 ± 0.77	−3.41	0.001 ***	−0.33
Self-esteem	19.3 ± 4.14	15.9 ± 5.23	3.70	0.000 ***	0.33
Confrontational	10.4 ± 2.63	9.9 ± 2.92	0.795	0.429	
Distancing	8.8 ± 3.04	8.02 ± 2.80	1.45	0.148	
Self-control	9.3 ± 2.85	10.1 ± 2.82	−1.28	0.201	
Social support	11.6 ± 3.19	10.6 ± 3.82	1.40	0.163	
Responsibility	6.5 ± 2.47	6.8 ± 2.70	−0.585	0.560	
Escape/avoidance	6.0 ± 3.62	8.8 ± 4.24	−3.45	0.001 ***	−0.33
Problem solving	11.8 ± 2.64	10.7 ± 3.30	1.91	0.059 *	0.18
Positive re-evaluation	14.4 ± 3.83	12.6 ± 3.8	2.35	0.020 *	0.22

* *p* ≤ 0.05, ** *p* ≤ 0.01, *** *p* ≤ 0.001.

**Table 5:** Differences and relation between women with and with no marital satisfaction and the types of violence perceived in their partner.

Types of Violence	Group 1 with Marital Satisfaction n = 43 Mean SD	Group 2 with Marital Dissatisfaction n = 61 Mean SD	*t* Value	Sig.	ƞ
Physical	1.00 ± 0.00	1.03 ± 0.12	−1.91|	0.060	
Economic	1.12 ± 0.28	1.55 ± 0.75	−4.04	0.000 ***	−0.35
Intimidation	1.04 ± 0.15	1.32 ± 0.57	−3.55	0.001 ***	−0.31
Psychological	1.09 ± 0.19	1.63 ± 0.86	−4.70	0.000 ***	−0.39
Control	1.17 ± 0.40	1.61 ± 0.93	−3.29	0.001 ***	−0.29
Humiliation/devaluation	1.01 ± 0.15	1.40 ± 0.65	−4.36	0.000 ***	−0.38
Blackmail	1.05 ± 0.10	1.58 ± 0.68	−5.97	0.000 ***	−0.47
Sexual	1.10 ± 0.23	1.41 ± 0.69	−3.29	0.002 ***	−0.28

*** *p* ≤ 0.001.

**Table 6:** Differences and relation between men with and with no marital satisfaction and their gender role, self-esteem and type of coping.

	Group 3 with Marital Satisfaction n = 58 Mean SD	Group 4 with Marital Dissatisfaction n = 46 Mean SD	*t* Value	Sig.	ƞ
Femininity	5.24 ± 0.83	4.75 ± 1.01	2.70	0.008 **	0.25
Masculinity	5.01 ± 0.77	4.85 ± 0.94	0.892	0.375	
Machismo	2.84 ± 0.81	3.40 ± 0.88	−3.37	0.001 **	−0.31
Submission	2.31| ± 0.66	2.72 ± 0.86	−2.75	0.007 **	−0.25
Self-esteem	21.0 ± 2.60	17.2 ± 4.62	4.99	0.000 ***	0.45
Confrontational	10.3 ± 2.97	10.3 ± 3.11	0.010	0.992	
Distancing	8.0 ± 2.92	8.4 ± 3.26	−0.687	0.494	
Self-control	11.0 ± 2.97	11.2 ± 3.85	−0.390	0.697	
Social support	10.8 ± 2.91	10.4 ± 3.56	0.625	0.533	
Responsibility	6.6 ± 1.90	6.7 ± 1.98	−0.241	0.810	
Escape/avoidance	5.0 ± 3.37	7.0 ± 3.96	−2.89	0.005 ***	0.54
Problem solving	13.1 ± 2.74	11.8 ± 3.07	2.29	0.024 *	0.44
Positive re-evaluation	14.0 ± 3.26	13.3 ± 4.04	0.983	0.328	

* *p* ≤ 0.05, ** *p* ≤ 0.01, *** *p* ≤ 0.001.

**Table 7:** Differences and relation between men with and with no marital satisfaction and the types of violence perceived in their partner.

Types of Violence	Group 3 with Marital Satisfaction n = 58 Mean SD	Group 4 with Marital Dissatisfaction n = 46 Mean SD	*t* Value	Sig.	ƞ
Physical	1.10 ± 0.05	1.17 ± 0.52	−2.16	0.036 *	−0.09
Economic	1.22 ± 0.42	1.83 ± 0.87	−4.29	0.000 *	−0.40
Intimidation	1.06 ± 0.18	1.45 ± 0.75	−3.37	0.001 ***	−0.33
Psychological	1.23 ± 0.45	1.84 ± 0.78	−4.67	0.000 ***	−0.43
Control	1.25 ± 0.40	2.23 ± 1.03	−6.07	0.000 ***	−0.53
Humiliation-devaluation	1.11 ± 0.26	1.57 ± 0.77	−3.83	0.000 ***	−0.37
Blackmail	1.12 ± 0.28	1.76 ± 0.78	−5.29	0.000 ***	−0.47
Sexual	1.09 ± 0.22	1.48 ± 0.71	−3.65	0.001 ***	−0.34

* *p* ≤ 0.05, *** *p* ≤ 0.001.

**Table 8:** Correlations between the studied variables and marital satisfaction in women.

Variable	Marital Satisfaction r
Gender role	
Femininity	0.338 **
Masculinity	0.247 *
Machismo	0.164
Submission	−0.320 **
Self-esteem	0.333 **
Coping Style	
Confrontational	0.078
Distancing	0.143
Self-control	−0.126
Social support	0.138
Responsibility	−0.058
Escape/avoidance	−0.323 **
Problem solving	0.186
Positive re-evaluation	0.227 *
Types of violence	
Physical	−0.157
Economic	−0.332 **
Intimidation	0.290 **
Psychological	−0.370 **
Control	−0.278 **
Humiliation/devaluation	−0.346 **
Blackmail	−0.447 **
Sexual	−0.266 **

* *p* ≤ 0.05, ** *p* ≤ 0.01.

**Table 9:** Correlations between the studied variables and marital satisfaction in men.

Variable	Marital Satisfaction r
Gender role	
Femininity	0.259 **
Masculinity	0.090
Machismo	−0.317 **
Submission	−0.263 **
Self-esteem	0.465 **
Coping	
Confrontational	0.001
Distancing	−0.068
Self- control	−0.039
Social support	0.062
Responsibility	−0.024
Escape/avoidance	−0.276 **
Problem solving	0.221 *
Positive re-evaluation	0.097
Type of violence	
Physical	−0.234 *
Economic	−0.415 **
Intimidation	−0.347 **
Psychological	−0.441 **
Control	−0.548 **
Humiliation/devaluation	−0.384 **
Blackmail	−0.498 **
Sexual	−0.371 **

* *p* ≤ 0.05, ** *p* ≤ 0.01.

**Table 10:** Structure matrix of the discriminant canonical functions: Women.

	Function 1
V Blackmail	0.639
V Psychological	0.509
V Humiliation-devaluation	0.472
Femininity	−0.460
Self-esteem	−0.451
V Economic	0.450
Escape-avoidance	0.437
Submission	0.433
V Intimidation	0.387
V Control	0.370
V Sexual	0.353
Masculinity	−0.325
Positive revaluation	−0.298

**Table 11:** Structure matrix of the discriminant canonical functions: Men.

	Function 1
V Control	0.728
V Blackmail	0.638
Self-esteem	−0.585
V Psychological	0.546
V Economic	0.508
V Humiliation/devaluation	0.463
V Sexual	0.444
V Intimidation	0.412
Escape/avoidance	0.319
Submission	0.303
Femininity	−0.298
V Physical	0.267
Problem solving	−0.252

## Data Availability

The link to the database is added to be consulted https://drive.google.com/file/d/1ndizrROn3jB23Xoi0yRWvH-60aRgLfns/view?usp=sharing (accessed on 24 April 2023).
